# The Ideal Cardiovascular Health Metrics Associated Inversely with Mortality from All Causes and from Cardiovascular Diseases among Adults in a Northern Chinese Industrial City

**DOI:** 10.1371/journal.pone.0089161

**Published:** 2014-02-24

**Authors:** Yan Liu, Hong-jie Chi, Liu-fu Cui, Xin-chun Yang, Yun-tao Wu, Zhe Huang, Hai-yan Zhao, Jing-sheng Gao, Shou-ling Wu, Jun Cai

**Affiliations:** 1 Department of Cardiology, Kailuan Hospital, Hebei United University, Tangshan, China; 2 Department of Cardiology, Chaoyang Hospital, Capital Medical University, Beijing, China; 3 Department of Rheumatism and Immunology, Kailuan Hospital, Hebei United University, Tangshan, China; FuWai hospital, Chinese Academy of Medical Sciences, China

## Abstract

**Background and Aims:**

The American Heart Association has recently established seven ideal cardiovascular health metrics for cardiovascular health promotion and disease reduction (i.e., non-smoking, normal body mass index, physically active, healthy diet, and normal levels of cholesterol, blood pressure and fasting blood glucose). The present study seeks to evaluate how well these metrics predict mortality from all causes and cardiovascular diseases in adult Chinese living in a northern industrial city.

**Methods and Results:**

Data of 95,429 adults who participated in the Kailuan cohort study from June 2006 to October 2007 was analyzed. All participants underwent questionnaire assessment, clinical examination, laboratory assessments and were followed up biannually. During a median follow-up of 4.02 years, 1,843 deaths occurred, with 597 deaths resulting from cardiovascular diseases. Lower mortality rates from all causes and cardiovascular diseases were observed among the subjects who met a higher number of the ideal health metrics. Compared to the participants who met none or one ideal health metric, those meeting ≥5 ideal health metrics had a lower risk of all-cause mortality by 30% (adjusted hazard ratio, 0.70; 95% confidence interval, 0.56–0.88) and a lower risk of mortality from cardiovascular diseases by 39% (adjusted hazard ratio, 0.61; 95% confidence interval, 0.41–0.89) . Four metrics (smoking status, physical activity, blood pressure and fasting blood glucose) were significantly associated with all-cause mortality. Three metrics (physical activity, blood pressure and fasting blood glucose) were significantly associated with mortality from cardiovascular diseases.

**Conclusion:**

The number of ideal health metrics is negatively associated with mortality rates from all causes and cardiovascular diseases among adults in a Northern Chinese industrial city. The data supports the AHA recommendation of ideal health metrics for adults from Northern China.

## Introduction

An impressive decline in the mortality rate from coronary heart disease and stroke over the past four decades has been achieved in developed and high-income countries [1], [2]. In the United States, the death rate from cardiovascular diseases (CVD) declined by 30.6% from 1998 to 2008 [3]. Recently, the American Heart Association (AHA) set a goal to improve the cardiovascular health of Americans by 20% until 2020. To measure progress toward this goal for cardiovascular health, the AHA has defined seven behaviors and risk factors (smoking status, body mass index, physical activity, healthy dietary score, total cholesterol, blood pressure, and fasting blood glucose) as health metrics and created three stages for each metric to reflect poor, intermediate, and ideal cardiovascular health status [4]. Although it has been reported that the number of ideal cardiovascular health metrics is a strong predictor of mortality from all causes and diseases of the circulatory system among American adults [5], there is limited information concerning the application of these metrics to adult Chinese.

Contrary to the United States, the proportional death rate due to CVD increased to approximately 32% in 2005 in China [6]. The coronary heart diseases death in adult Chinese (35–84 years old) is predicted to increase dramatically by 64% during the decade 2020–2029, compared with 2000–2009 [7]. The concept of primordial prevention advocated by the AHA is also important in controlling the increasing mortality rate from CVD in the Chinese population. Based on a large population of a community in a northern Chinese industrial city, we previously reported that only 0.1% study population met all seven ideal cardiovascular health metrics; furthermore, for every increment in the number of ideal cardiovascular health metrics met, the risk of myocardial infarction and stroke declined 16% [8]. However, little is known about whether the new AHA 2020 metrics can predict mortality rates from all causes and CVD in the Chinese population. Thus, we conducted a study on the same population to investigate the reliability of the AHA metrics as predictors for all-cause and CVD mortality in Chinese adults.

## Methods

### Study Design and Participants

The design of Kailuan study has been described previously [8]. In brief, it is a prospective cohort study based on the Kailuan community in Tangshan City, which is an industrial and modern city located in the central section of the circulating Bohai Sea Gulf region of China. From June 2006 to October 2007, the health records of 101,510 residents (81,110 males and 20,400 females, 18–98 years old) of Kailuan community were built up in eleven local hospitals that are responsible for healthcare of the community. All residents who met the following criteria were recruited into the Kailuan study: (1) aged 18 years or older; (2) provided informed consent; (3) updated their health status according to the follow-up protocol. All participants underwent questionnaire assessment, clinical examination, and laboratory assessments. Specially trained doctors and nurses performed all measurements, using standard protocols [8], [9], [10]. Participants with incomplete data were excluded. Ongoing evaluations included biannual measurement of laboratory parameters and recording of adverse events. The study followed the guidelines of the Helsinki Declaration and was approved by the Ethics Committees of Kailuan General Hospital, Tiantan Hospital and Beijing Chaoyang Hospital. Written informed consent was obtained from all participants.

### Questionnaire Assessment

Research doctors administered questionnaires in person. Smoking status was self-reported and classified as “never,” “former,” or “current”. Physical activity was evaluated from responses to questions on the type and frequency of physical activity at work and during leisure time. Salt intake was classified as “low,” “medium,” or “high” based on responses to questions related to salt preferences. We used “low” salt intake as a surrogate of ideal diet in this study as salted food intake is a serious issue in China. The questionnaires provided an approximation of whether an individual's diet was “ideal,” “intermediate,” or “poor”. A history of myocardial infarction (MI), stroke, or cancer was defined as any self-reported previous physician diagnosis of MI, stroke, or cancer. The use of antihypertensive, cholesterol-lowering, and glucose-lowering medications within the past two weeks before the baseline interview was self-reported. The average monthly income of each family member was reported as “<¥600,” “¥600–1,000,” or “≥¥1,000”. The education level was categorized as “none or primary school,” “middle school,” or “college or university”. Alcohol use was defined as drinking alcohol more than 100 ml per day at least for 1 year.

### Anthropometric Measurements

Anthropometric indices included height and weight. Height was measured to the nearest 0.1 cm using a tape measure, and weight was measured to the nearest 0.1 kg using calibrated platform scales. Body mass index (BMI) was calculated as body weight (kg) divided by the square of height (m^2^).

### Blood Pressure Measurement

Blood pressure (BP) was measured on the left arm to the nearest 2 mm Hg using a mercury sphygmomanometer. After participants had rested for at least 5 minutes, two readings each of systolic and diastolic blood pressure (SBP and DBP, respectively) were taken at a 5-minute interval. The average of the 2 readings was used for data analysis. If the two measurements differed greater than 5 mm Hg, an additional reading was taken, and the average of the three readings was used for data analysis. Because blood pressure was measured by mercury sphygmomanometer in this study, we calculated constitute of end digits of systolic blood pressure and diastolic blood pressure to test if end digits preferences exist.

### Laboratory Assessments

Blood samples were collected from the antecubital vein in the morning after an overnight fast, and were analyzed within 4 hours. Fasting blood glucose (FBG) was measured with the hexokinase/glucose-6-phosphate-dehydrogenase method. Total cholesterol (TC) and triglycerides were measured enzymatically (inter-assay coefficient of variation <10%; Mind Bioengineering Co. Ltd., Shanghai, China). All biochemical variables were measured with an autoanalyzer (Hitachi 747; Hitachi, Tokyo, Japan) at the central laboratory of Kailuan General Hospital.

### Cardiovascular health metrics

Participants who had never smoked were defined as showing evidence of ideal health. Former smokers were defined as showing evidence of intermediate health. Participants who were currently smoking were defined as showing evidence of poor health. Participants with a BMI of ≥30, 25 to <30, and <25 kg/m^2^ had poor, intermediate, and ideal health, respectively. For physical activity, ideal health, intermediate health, and poor health were defined as ≥80, 1 to 79, and 0 minutes of moderate or vigorous activity per week, respectively. As a surrogate of ideal diet, low salt intake was considered as ideal health state, participants with intermediate salt intake were assigned to intermediate health, and those with high salt intake were assigned to poor health. Ideal health, intermediate health, and poor health for TC were defined as <200 mg/dL, 200–239 mg/dL, and ≥240 mg/dL without treatment. Participants taking lipid-lowering agents were identified as intermediate health if they had ideal TC level and as poor health if they had intermediate level. For participants without treatment for hypertension, SBP <120 mm Hg and DBP <80 mm Hg were defined as ideal health, SBP of 120 to 139 mm Hg and DBP of 80 to 89 mm Hg were defined as intermediate health, and SBP ≥140 mm Hg or DBP ≥90 mm Hg were defined as poor health, respectively. Participants who were being treated for hypertension were identified as intermediate health if they had ideal BP level and as poor health if they had intermediate BP level. For participants with no treatment for hyperglycemia, ideal health, intermediate health, and poor health were defined as fasting plasma glucose <100 mg/dL, 100–125 mg/dL, and ≥126 mg/dL, respectively. Participants who were taking hypoglycemic agents were identified as “intermediate” if they had ideal FBG level, and as “poor” if they had intermediate FBG level.

### Follow up and Death Certification

All participants were followed up from the baseline examination between June 2006 and October 2007 through December 31, 2010 or to the date of death or loss to follow-up, with death as the endpoint event. Death and the cause of death were ascertained by professional doctors through surveying discharge lists from local hospitals and death certificates from state vital statistics offices and by contacting participants for survival status twice a year. Death from all causes was defined as death caused by any factor during the follow-up period. The International Statistical Classification of Diseases and Related Health Problems, 10th revision, codes I05–I09, I11, I20–I27, and I30–I52, were used to identify deaths from diseases of cardiovascular system.

### Statistical Analyses

Statistical analyses were performed using SAS software, version 9.2 (SAS Institute, Cary, North Carolina, USA). Continuous variables were described as mean±standard deviation (SD) and compared using a two sample Student's *t-*test. Categorical variables were described by percentages and compared using χ^2^ test. The prevalence of each baseline cardiovascular health metric or their combinations was computed. Mortality rates per 1000 person-years (PY) of follow-up were calculated. Adjustment for age and sex was performed by use of the direct method with the year 2006 China population [11]. Cox proportional hazards regression analysis was used to estimate the risk for mortality for individual and number of health metrics. The hazard ratios were expressed as per 1 standard deviation increased value. The analyses for risk estimates were performed with adjustment for age, sex, average income, education level, alcohol use, history of MI, history of stroke, and history of cancer. All statistical tests were 2-sided, and *P*<0.05 was considered statistically significant.

## Results

### Baseline Characteristics and the Prevalence of Ideal Cardiovascular Health Metrics

**Figure 1:**
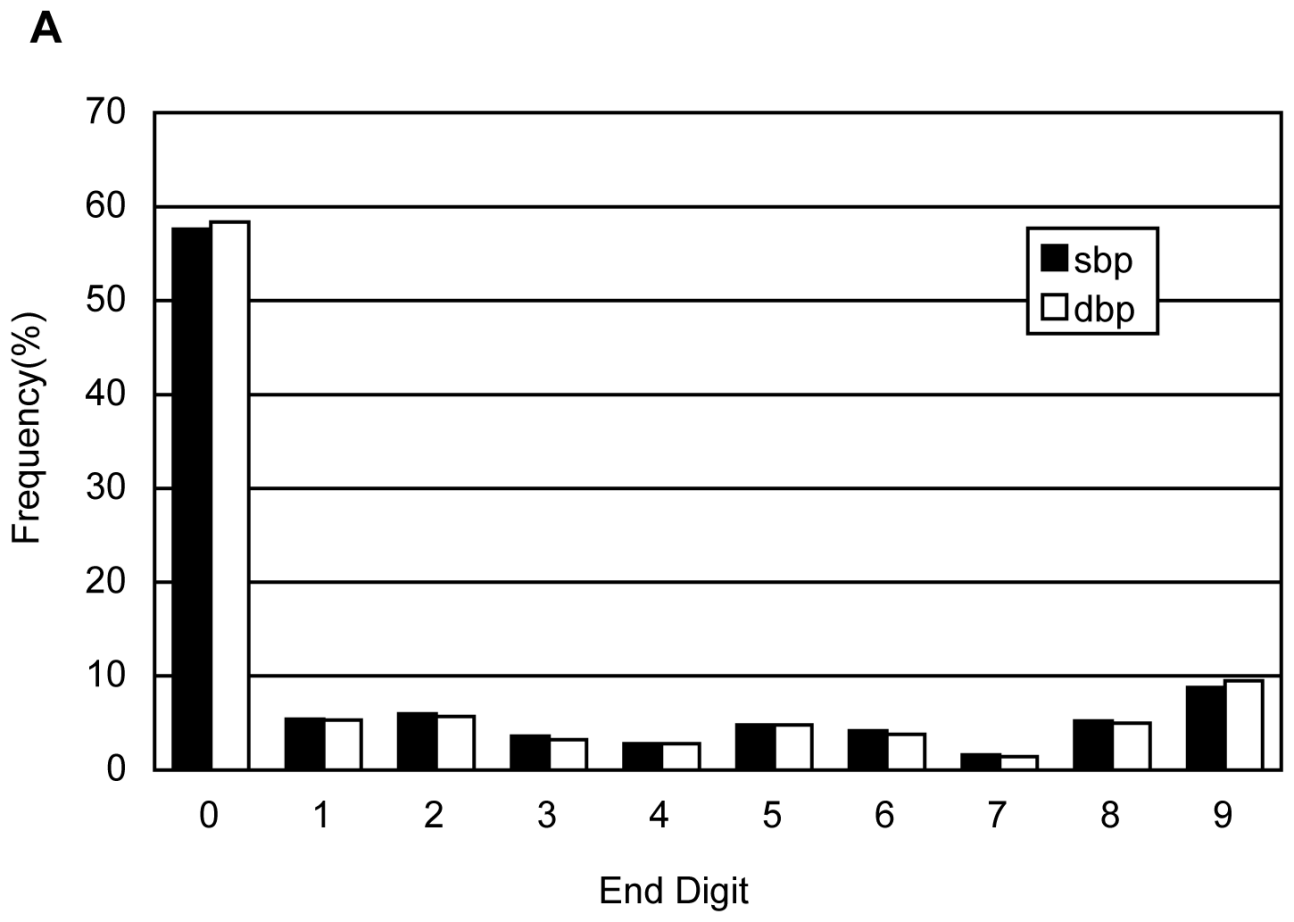
Distribution of end digits of systolic blood pressure and diastolic blood pressure. SBP indicates systolic blood pressure; DBP, diastolic blood pressure.

Of the 101,510 participants of Kailuan study, 6,081 had incomplete information for the seven cardiovascular health behaviors and factors and were excluded. Analyses were confined to the remaining 95,429 participants (94% of the cohort), which included 76,109 males and 19,320 females. The average age of the remaining population was 51.46±12.46 years. **Table 1** and **Table 2** shows baseline characteristics of total participants. **Table 1** compared the baseline characteristics between the survivors and the deceased. **Table 2** compared the baseline characteristics by the number of cardiovascular metrics. The survivors tended to be younger, had lower levels of SBP, DBP and FBG, had lower incidences of previous MI, stroke, and cancer, and had higher incidence of ideal smoking status, ideal BP and ideal FBG. The survivors also had a higher average income. By contrast, the deceased had lower levels of TC and BMI, were better educated, and had higher incidence of ideal physical activity and ideal diet. All of the above differences were statistically significant (*P*<0.05). The participants with more ideal health metrics were younger, had lower levels of SBP, DBP, TC, FBG, BMI, and had lower incidences of alcohol use, previous MI, stroke, and cancer (**Table 2**). The distribution of end digits of SBP and DBP was shown in **Figure 1**. Fifty to sixty percent of measurements of SBP and DBP were ended with zero.

**Table 1:** Baseline Characteristics of Participants.

Characteristics	Total (n = 95429)	Survivors (n = 93586)	Deceased (n = 1843)	*P* values
Age, years	51.46±12.46	51.22±12.36	63.51±11.71	<.0001
Men, N (%)	76109 (79.75)	74406 (79.51)	1703 (92.40)	<.0001
SBP, mm Hg	131.00±21.09	130.80±20.97	141.80±23.98	<.0001
DBP, mm Hg	83.56±11.80	83.52±11.77	85.84±13.33	<.0001
TC, mg/ml	191.69±44.84	191.70±44.73	189.20±50.03	0.0289
FBG, mg/ml	99.27±30.47	99.11±30.14	107.30±43.63	<.0001
BMI, kg/m^2^	25.05±3.49	25.05±3.49	24.83±3.81	0.0112
Income, ¥/month*, N (%)				
<¥600	27622 (28.97)	27016 (28.89)	606 (32.95)	0.0004
¥600–1000	61333 (64.33)	60205 (64.39)	1128 (61.34)	
≥¥1000	6389 (6.70)	6284 (6.72)	105 (5.71)	
Education level, N (%)				
No/primary	10381 (10.89)	514 (27.93)	9867 (10.55)	<.0001
Middle school	78386 (82.20)	1273 (69.18)	77113 (82.46)	
College/university	6593 (6.91)	53 (2.88)	6540 (6.99)	
Alcohol use, N (%)				
yes	17123 (17.95)	16812 (17.97)	311 (16.88)	0.2271
History of MI, N (%)				
yes	1282 (1.34)	1186 (1.27)	96 (5.21)	<.0001
History of stroke, N (%)				
yes	2490 (2.61)	2281 (2.44)	209 (11.35)	<.0001
History of cancer, N (%)				
yes	364 (0.39)	350 (0.38)	14 (0.78)	0.0071
Ideal smoking status, N (%)	56924(59.65)	55907(59.74)	1017(55.18)	<.0001
Ideal BMI, N (%)	49160(51.51)	48176(51.48)	984(53.39)	0.104
Ideal physical activity, N (%)	15005(15.72)	14629(15.63)	376(20.4)	<.0001
Ideal diet, N (%)	8866(9.29)	8664(9.26)	202(10.96)	0.013
Ideal BP, N (%)	19013(19.92)	18816(20.11)	197(10.69)	<.0001
Ideal FBG, N (%)	65278(68.4)	64149(68.55)	1129(61.26)	<.0001
Ideal TC, N (%)	57069(59.8)	55976(59.81)	1093(59.31)	0.66

SBP indicates systolic blood pressure; DBP, diastolic blood pressure; FBG, fasting blood glucose;

TC, total cholesterol; BMI, body mass index; MI, myocardial infarction; BP, blood pressure.

*Average monthly income of the family member.

**Table 2:** Baseline Characteristics by Number of Ideal Cardiovascular Health Metrics.

Characteristics	No. of Ideal Cardiovascular Health Metrics					*P* values
	0–1	2	3	4	≥5	
n	13659	23828	29095	20284	8563	
Age, years	51.86±10.656	52.54±11.396	52.09±12.44	50.79±13.468	47.25±14.472	<0.001
Men, N (%)	12645(92.6)	20628(86.6)	23607(81.1)	14711(72.5)	4518(52.8)	<0.001
SBP, mm Hg	139.52±19.964	136.27±19.806	132.36±20.095	124.83±19.829	112.7±17.359	<0.001
DBP, mm Hg	88.9±11.322	86.71±11.049	84.21±10.999	79.91±10.93	72.7±9.48	<0.001
TC, mg/ml	222.75±42.466	202.66±45.773	185.91±43.476	175.52±37.2	169.55±30.071	<0.001
FBG, mg/ml	119.72±40.336	105.54±34.951	95.07±25.552	89.45±17.893	86.69±12.464	<0.001
BMI, kg/m^2^	27.49±2.861	26.34±3.307	24.97±3.364	23.21±2.802	22.19±2.337	<0.001
Income, ¥/month*, N (%)						
<¥600	1731(12.7)	2829(11.9)	3105(10.7)	1968(9.7)	748(8.7)	<0.001
¥600–1000	11196(82.1)	19817(83.2)	24333(83.7)	16706(82.4)	6334(74)	
≥¥1000	717(5.3)	1163(4.9)	1641(5.6)	1597(7.9)	1475(17.2)	
Education level, N (%)						
No/primary	4944(36.2)	7314(30.7)	7898(27.2)	5128(25.3)	2338(27.3)	<0.001
Middle school	7711(56.5)	15022(63.1)	19458(66.9)	13819(68.2)	5323(62.2)	
College/university	986(7.2)	1469(6.2)	1714(5.9)	1327(6.5)	893(10.4)	
Alcohol use, N (%)						
yes	4341(31.8)	5298(22.3)	4564(15.7)	2243(11.1)	677(7.9)	<0.001
History of MI, N (%)						
yes	289(2.1)	398(1.7)	338(1.2)	185(0.9)	72(0.8)	<0.001
History of stroke, N (%)						
yes	520(3.8)	706(3)	746(2.6)	387(1.9)	131(1.5)	<0.001
History of cancer, N (%)						
yes	56(0.4)	83(0.4)	119(0.4)	80(0.4)	26(0.3)	0.541

SBP indicates systolic blood pressure; DBP, diastolic blood pressure; FBG, fasting blood glucose; TC, total cholesterol; BMI, body mass index; MI, myocardial infarction; BP, blood pressure.

*Average monthly income of the family member.

### Mortality due to all causes and due to cardiovascular diseases

During a median follow-up of 4.02 years, 1,843 deaths (aged 63.51±11.71 years) occurred, which included 1,703 males (aged 63.72±11.79 years) and 140 females (aged 60.93±10.44 years). Among them, 457 died of cancers, 14 participants died of accidents, 597 died of cardiovascular diseases and 775 died of other reasons. The standardized mortality rate from all causes for all participants was 2.74/1000 PY, comparable to the standardized mortality rate of 3.17/1000 PY in the east region of China [12]. Due to the small numbers of participants with 0, 6, or 7 ideal health metrics, we pooled those with 0 or 1 ideal health metrics into one group that was labeled as 0 to 1 group, and those with 5, 6, or 7 ideal health metrics into another group that was labeled as ≥5 group. For participants with 0 to 1, 2, 3, 4, or ≥5 ideal health metrics, all-cause mortality rate was 3.48, 3.18, 2.87, 2.41, and 2.2 per 1000 PY and mortality rate from CVD was 1.59, 1.48, 0.86, 0.59, and 0.69 per 1000 PY, respectively. Of the 93 participants who met all seven health metrics at baseline, only one died over the follow-up period. A strong inverse association was observed between the number of ideal health metrics and the age- and sex- adjusted mortality rates from all causes or from CVD (**Table 3**).

**Table 3:** Sample Sizes, Rates, and Hazard Ratios for Mortality from All Causes and cardiovascular Diseases.

	No. of Ideal Cardiovascular Health Metrics				
	0–1	2	3	4	≥5
All participants(n = 95429)					
All causes					
Death/at risk, n	290/13659	507/23828	567/29095	369/20284	110/8563
age- and sex- adjusted rate/1000PY	3.48	3.18	2.87	2.41	2.20
Adjusted hazard ratios (95%CI) *	1.00	0.94(0.81–1.09)	0.85(0.74–0.99)	0.82(0.70–0.96)	0.70(0.56–0.88)
Cardiovascular diseases					
Death/at risk, n	110/13659	179/23828	175/29095	99/20284	34/8563
age- and sex- adjusted rate/1000PY	1.59	1.48	0.86	0.59	0.69
Adjusted hazard ratios (95%CI) *	1.00	0.88(0.69–1.12)	0.71(0.55–0.90)	0.58(0.44–0.77)	0.61(0.41–0.89)
Limited to participants without history of MI or stroke(n = 91698)					
All causes					
Death/at risk, n	223/12882	430/22776	492/28003	328/19684	91/8353
age- and sex- adjusted rate/1000PY	2.96	2.86	2.72	2.33	2.02
Adjusted hazard ratios (95%CI) *	1.00	0.99(0.84–1.17)	0.91(0.77–1.07)	0.86(0.73–1.03)	0.69(0.54–0.89)
Cardiovascular disease					
Death/at risk, n	75/12882	133/22776	142/28003	83/19684	25/8353
age- and sex- adjusted rate/1000PY	1.27	1.14	0.77	0.53	0.56
Adjusted hazard ratios (95%CI) *	1.00	0.89(0.67–1.19)	0.76(0.57–1.01)	0.60(0.44–0.83)	0.58(0.37–0.92)

PY indicates person-year; MI, myocardial infarction.

Mortality rates were calculated by direct method with the year 2006 population to adjust for age and sex.

*adjusted for age, sex, average income, education level, alcohol use, history of MI and stoke (limited to all participants), history of cancer.

After adjustment for age, sex, education level, average income, alcohol use, history of MI, stroke, and cancer, the hazard ratios (HR) and 95% confidence interval (CI) for all-cause mortality among participants with 2, 3, 4 or ≥5 ideal health metrics were 0.94 (0.81–1.09), 0.85 (0.74–0.99), 0.82 (0.70–0.96), and 0.70 (0.56–0.88) compared with participants with 0 to 1 ideal health metrics (**Table 3**). The HR and 95% CI for mortality from CVD of participants with 2, 3, 4 or ≥5 ideal health metrics were 0.88 (0.69–1.12), 0.71 (0.55–0.90), 0.58 (0.44–0.77), and 0.61 (0.41–0.89) compared with participants with 0 to 1 ideal health metrics (**Table 3**). Furthermore, after the exclusion of participants with history of MI or stroke, the strong inverse association between the number of ideal health metrics and the mortality rates from all causes or from CVD remained evident (**Table 3**).

After adjustment for age, sex, average income, education level, alcohol use, history of MI, history of stroke, and history of cancer, four metrics (smoking status, physical activity, BP, and FBG) were significantly associated with all-cause mortality (**Table 4**). These metrics also showed evidence of a graded response (all *P* values for trend <0.05), indicating that ideal health categories for these metrics were associated with a greater reduction in all-cause mortality than intermediate categories. Three metrics (physical activity, BP and FBG) were significantly associated with mortality from CVD (**Table 4**). The hazard ratios for mortality from CVD generally were similar with those for all-cause mortality in different categories for every metric.

**Table 4:** Hazard Ratios (95% Confidence Interval) for Mortality from All Causes and Cardiovascular Diseases for Different Categories of Health metrics.

	All–cause mortality*				Mortality from Cardiovascular Diseases*			
	Poor	Intermediate	Ideal	*P* values for trend	Poor	Intermediate	Ideal	*P* values for trend
Smoking status	1.00	1.00 (0.85–1.18)	0.89 (0.79–1.01)	0.048	1.00	0.95 (0.71–1.26)	0.88 (0.71–1.08)	0.359
BMI	1.00	0.86 (0.72–1.02)	1.00 (0.84–1.19)	0.092	1.00	0.91 (0.68–1.22)	0.83 (0.62–1.11)	0.160
Physical activity	1.00	0.81 (0.69–0.96)	0.65 (0.55–0.79)	<.001	1.00	0.92 (0.68–1.24)	0.67 (0.48–0.93)	0.002
Diet	1.00	1.07 (0.92–1.25)	1.12 (0.92–1.37)	0.261	1.00	1.09 (0.82–1.43)	1.31 (0.93–1.84)	0.138
BP	1.00	0.78 (0.71–0.87)	0.75 (0.64–0.88)	<.001	1.00	0.61 (0.50–0.74)	0.59 (0.43–0.80)	<.001
FBG	1.00	0.60 (0.52–0.70)	0.58 (0.51–0.66)	<.001	1.00	0.65 (0.50–0.84)	0.62 (0.49–0.78)	<.001
TC	1.00	1.00 (0.85–1.17)	1.05 (0.90–1.22)	0.295	1.00	0.93 (0.71–1.22)	0.91 (0.70–1.17)	0.149

BMI indicates body mass index; BP, blood pressure; FBG, fasting blood glucose; TC, total cholesterol;

*adjusted for age, sex, average income, education level, alcohol use, history of myocardial infarction, history of stroke, and history of cancer.

Furthermore, we analyzed the all-cause and cardiovascular mortality risk associated with individual risk factors as a continuous variable when applicable (**Table 5**). After adjustment for age, sex, average income, education level, alcohol use, history of MI, history of stroke, and history of cancer, four metrics (BMI, SBP, FBG and TC) were significantly associated with all-cause mortality (*P*<0.05), SBP and FBG were significantly associated with mortality from CVD (*P*<0.05).

**Table 5:** Hazard Ratios (95% Confidence Interval) for Mortality from All Causes and Cardiovascular Diseases for individual risk factors as a continuous variable.

	Standard Deviation	All–cause mortality*	*P* values	Mortality from Cardiovascular Diseases*	*P* values
BMI	3.49 kg/m^2^	0.92(0.88–0.97)	<.001	1.00(0.92–1.09)	0.966
SBP	21.09 mmHg	1.15(1.09–1.23)	<.001	1.27(1.15–1.40)	<.001
DBP	11.80 mmHg	1.03(0.98–1.10)	0.256	1.08(0.98–1.19)	0.131
FBG	30.47 mg/ml	1.19(1.14–1.25)	<.001	1.18(1.09–1.27)	<0.001
TC	44.84 mg/ml	0.94(0.90–0.99)	0.009	0.97(0.90–1.05)	0.465

BMI indicates body mass index; SBP, systolic blood pressure; DBP, diastolic blood pressure; FBG, fasting blood glucose; TC, total cholesterol;

Hazard ratios expressed per 1 standard deviation increased value.

*adjusted for age, sex, average income, education level, alcohol use, history of myocardial infarction, history of stroke, and history of cancer.

## Discussion

After the AHA defined a set of ideal cardiovascular health metrics to measure the progress toward the 2020 Impact Goal, several studies attempting to estimate the prevalence of ideal cardiovascular health in the United States have been published [5], [13], [14]. These studies have reported that the prevalence of all seven ideal cardiovascular health metrics in the US adult population was nearly 1% and that the proportion of adults who met five to seven ideal health metrics was nearly 20%, which indicated that few adults in the United States achieved ideal cardiovascular health. Our previous study has shown that only 0.1% participants met all seven ideal cardiovascular health behaviors and factors, and only 9.1% met ≥5 ideal health metrics [8]. Consistent with our results, another study in a Chinese population reported that only 52 of 9,962 participants (0.5%) had ideal levels of all seven health metrics and only 26.9% had ≥5 ideal health metrics [15]. What is clear from our data and previous studies is that most adults have poor cardiovascular health, and few have ideal cardiovascular health, both in China and the United States.

Ford and his colleagues were the first to report the relationship between the AHA 2020 goals and mortality rates from all causes and from CVD in the United States [5]. Their results demonstrated that mortality from all causes and from CVD showed declining trends with increasing number of ideal cardiovascular health behaviors and factors met in the adults of the United States. To our knowledge, our study is the first to report that this kind of strong inverse relationship between the number of cardiovascular health metrics and mortality from all causes and from CVD also are present in a population outside of the United States. Our previous work [8] and another prospective study [14] reported an inverse relationship between the cumulative incidence of CVD and the number of ideal health metrics, which indicates that the AHA metrics also reflect the subsequent risk of CVD. Above data suggests that attainment of the AHA 2020 goals could result in substantial reductions both in morbidity and mortality of CVD. The development of the AHA 2020 goals that emphasize cardiovascular health represents an important evolution from disease management to health promotion.

Controlling for confounding factors constitutes a major challenge in observational studies. In our study, the deceased tended to be older, had lower income level, were more commonly male, and more often had a history of MI, stroke, or cancer. To control various possible confounders, Cox proportional hazards model was used. After adjusting for the factors mentioned above, there continued to be a declining trend in the mortality from all causes and from CVD with increasing number of ideal health metrics. This kind of trend was still present after exclusion of participants with history of MI or stroke. Compared with participants with 0 to 1 ideal health metric, those with ≥5 metrics had a 30% lower risk of all-cause mortality and a 39% lower risk of mortality from CVD. Our results are consistent with those of a previous study in the United States in which Ford and his colleagues found that, compared with participants who have no ideal health metric, those having ≥5 metrics had a 78% reduction for risk of all-cause mortality and a 88% reduction for risk of mortality from CVD [5].

Four metrics (smoking status, physical activity, BP, and FBG) showed a gradient in risks for mortalities, suggesting that when adults move from poor to intermediate health and when they move from intermediate to ideal health, overall health gains are feasible. Not all of the individual metrics contributed to the reduction of mortality risks. In our study, low-salt diet was not significantly related to mortality from all causes or from CVD. The reasons are not clear. Of note, the AHA used healthy dietary score, which was calculated based on the consumption of grains, fruits, vegetables, dairy, meats, and salt, to evaluate cardiovascular health, and one study demonstrated that healthy dietary score was significantly associated with mortality in adults of the United States [16]. The information on salt intake in the current study was self-reported form the questionnaire survey and was not a real measurement and “low” salt intake alone was used as a surrogate of ideal diet, these factors mentioned above possibly weakening the effects of diet on mortality.

Our results should be considered in light of several limitations. First, the median follow-up time was only 4.02 years, which may not be long enough to sufficiently evaluate endpoint events. Second, we did not adhere perfectly to all of the AHA 2020 health metrics for practical reasons. For example, we used “low” salt intake as a surrogate of ideal diet, which may negate the effects of diet on mortality. Third, there was a significant difference in sex distribution, which could lead to selection bias. However, we calculated age- and sex- adjusted mortality rates to diminish the influences of these differences on mortality. Fourth, blood pressure was measured by mercury sphygmomanometer in this study and the end digit preferences exist. That is to say, there were some measurement errors in our study. Because the measurement errors belong to systematic error, which could not be reduced by large sample size. Therefore, the misclassification could not be avoided. Fifth, because of the limitation for information collection, we only provided the final mortality for cardiovascular diseases and did not supply the number of death for specific cardiovascular diseases. Lastly, we only used a single measurement for all the health metrics; thus, we were unable to account for changes in the metrics that may have occurred during the follow-up period.

Overall, our findings add to the previous report that demonstrates the inverse strong relationship between the AHA 2020 goals and all-cause mortality and mortality from CVD. It is the first time to prove that this relationship is also present in a Northern Chinese cohort. Our findings suggest that to reduce all-cause mortality and mortality from CVD and promote cardiovascular health in the adult population of China, individuals, communities and health-care providers need to be integrated to focus on primordial prevention of unhealthy lifestyles. Considering China's large population, if there is no action to address this public health issue, a growing Chinese population with non-ideal cardiovascular behaviors and risk factors will increase the burden of public health in China.
